# Exposure to Second-Hand Smoke in Public Places and Barriers to the Implementation of Smoke-Free Regulations in The Gambia: A Population-Based Survey

**DOI:** 10.3390/ijerph18126263

**Published:** 2021-06-09

**Authors:** Bai Cham, Noreen Dadirai Mdege, Linda Bauld, John Britton, Umberto D’Alessandro

**Affiliations:** 1 Disease Control and Elimination Theme, Medical Research Council Unit The Gambia at the London School of Hygiene and Tropical Medicine, Atlantic Road, Fajara, P.O. Box 273 Banjul, The Gambia; udalessandro@mrc.gm; 2 Department of Health Sciences, University of York, York YO10 5DD, UK; noreen.mdege@york.ac.uk; 3 Usher Institute and SPECTRUM Consortium, College of Medicine and Veterinary Medicine, University of Edinburgh, Edinburgh EH 8 9AG, UK; Linda.Bauld@ed.ac.uk; 4 UK Centre for Tobacco and Alcohol Studies, Division of Epidemiology and Public Health, University of Nottingham, Nottingham NG5 1PB, UK; j.britton@outlook.com

**Keywords:** smoking, second-hand smoke, The Gambia, smoke-free regulations

## Abstract

Introduction: Second-hand smoke is associated with more than 1.2 million deaths per year among non-smokers. Smoking in public places is prohibited in The Gambia but there is no information on the level of exposure to second-hand smoke among adolescents and adults 15–64 years. The aim of this study was to assess the level and predictors of exposure to second-hand smoke in public places and compliance with smoke-free regulations in The Gambia. Methods: A population-based survey was conducted in an established Health and Demographic Surveillance System (HDSS). A total of 4547 participants (15–64 years) from households within the Farafenni HDSS were interviewed at their homes but only 3343 were included in our analysis. Factors associated with exposure to second-hand smoke in public places were assessed by three different multivariable regression models. Results: Exposure to tobacco smoke in public places was high (66.1%), and higher in men (79.9%) than women (58.7%). Besides being male, less education, lower household income, urban residence and not aware of smoke-free regulations were strongly associated with exposure to second-hand smoke. Conclusion: Despite existing smoke-free regulations, reported exposure to second-hand smoke remains high in public places in The Gambia. The Ministry of Health should continue to strengthen their advocacy and sensitization programs to ensure smoke-free regulations are fully implemented. Some population subgroups are at a higher risk of exposure and could be targeted by interventions; and settings where these subgroups are exposed should be targeted by enforcement efforts.

## 1. Introduction

Exposure to second-hand tobacco smoke (hereafter referred to as second-hand smoke) is associated with more than 1.2 million deaths per year worldwide among non-smokers; about 11 million disability-adjusted life years (DALYs) are lost due to second-hand smoke exposure, with 61% of them occurring among children [1]. In 2004, 40% of children, 33% of men and 35% of women in 192 countries were exposed to second-hand smoke indoors [1]. There is no safe level of exposure to tobacco smoke [2]. Article 8 of the World Health Organization Framework Convention on Tobacco Control (WHO FCTC) recommends eliminating exposure to tobacco smoke in workplaces and public places, both indoor and outdoor, and this includes public transport [2]. Article 20 (Research, surveillance, and exchange of information) stresses the importance of surveillance for assessing the tobacco epidemic, monitoring tobacco use and the related prevention policies [2].

The Gambia’s 2017 Global Youth Tobacco Survey (GYTS) found that 61.8% of students aged 13–15 years were exposed to second-hand smoke in enclosed public places [3]. A recent survey in secondary schools also reported a high prevalence of second-hand smoke exposure among young people aged 12–20 years in The Gambia: 59.2% for indoor public places and 61.4% for outdoor public places [4]. There are several regulations and policies on tobacco control in The Gambia, including the Prohibition of Smoking in Public Places Act [5]. The Tobacco Control Act 2016 was adopted in December 2016 and it came into force in July 2018 [6]. The Tobacco Control Regulations 2019 [7], which supports the enforcement of the Tobacco Control Act 2016, was signed and gazetted in August 2019. Based on the existing international treaties and local regulations, the country has the legal obligation and instruments to protect the public from exposure to tobacco smoke. However, despite the numerous achievements in tobacco control as highlighted above, there are still gaps especially on the implementation of smoke-free regulation in The Gambia. Article 20 of the WHO FCTC (Research, surveillance, and exchange of information) stresses the importance of surveillance for assessing the tobacco epidemic, monitoring tobacco use and the related prevention policies including those for the protection of the public and non-smokers, and the level of compliance and the barriers to the implementation of the tobacco control regulations [2]. The aim of this study was to address this critical evidence gap by assessing, for the first time, the level and predictors of exposure to second-hand smoke in indoor and outdoor public places among both adolescents and adults, and compliance with smoke-free regulations in The Gambia.

## 2. Materials and Methods

### 2.1. Study Setting and Design

The study was conducted in The Gambia from 20 January to 26 March 2020 as part of the Farafenni Health and Demographic Surveillance System (HDSS) which was established in October 1981 by the Medical Research Council Unit The Gambia (MRCG) and is updated every four months [8]. The HDSS is in the North Bank Region about 170 km from the capital, Banjul; includes the provincial town of Farafenni and 22 peri-urban and 40 rural villages; and covered a population of 59,910 individuals at the time of data collection. Farafenni town and the peri-urban villages were included in July 2002 and designated the “urban demographic surveillance area” [8]. All residents are part of the HDSS except the military staff, students attending boarding schools and staff of the Ministry of Health from other regions but in the North Bank Region on postings. Entry to the HDSS is through initial enumeration, birth, or immigration [8]. Individuals 15–64 years old were randomly selected from the HDSS database and interviewed at home.

### 2.2. Study Instrument/Questionnaire

In addition to the HDSS routine information (age, gender, education, marital status, ethnicity, and residence), household income, current and past smoking status, awareness of the tobacco control regulations, exposure to second-hand smoke and several other variables (not included in this analysis) were also collected. We adapted questions from the tobacco component of the WHO STEP survey questionnaire [9], as well as the Global Adults Tobacco Survey (GATS) questionnaire [10]. The questionnaire was translated into Wolof and Mandinka, the two main local languages, and then pilot-tested. Interviews were carried out by trained field workers and data electronically captured using the Research Electronic Data Capture (REDCap; https://projectredcap.org/software/ (accessed on 28 November 2019)) with tablets and mobile phones programmed with the data collection app.

### 2.3. Sampling Framework and Sample Size

The target population, i.e., 15–64-year-old individuals, represented about half (51.1%) of the total population covered by the Farafenni HDSS. The sample size was estimated assuming the prevalence of smoking was 20%, based on a national prevalence of 15.6% [11], a margin of error of 5%, and a design effect of 1.5 [9]. In addition, since the response rate from the last national STEP survey in The Gambia was 79%, and to adjust for non-response and increase the power, the sample size was increased by 20%. This resulted in a sample size of 4613 which was increased to 6000 to allow for emigration and non-response. 

A multistage sampling technique was applied using the three levels of residence within the HDSS (urban, semi-urban, and rural). At the first stage, a total of 42 urban, semi-urban and rural villages were randomly selected. Stage two involved sampling of households within the selected villages. There are 3727 compounds and 6037 households within the Farafenni HDSS. The average household size is 10 and, on average, there are five 15–64- year-old individuals per household; a total of 2532 households were randomly selected from the HDSS database. In stage three, one to five people between the ages of 15–64 years were initially randomly selected per household proportional to the size of the household, for a total of 6000. Due to seasonal migration, some selected individuals were unavailable. Therefore, one month after starting the data collection process, 2856 additional individuals were randomly selected, resulting a total sample of 8856 individuals. We were only able to interview 4547 but our analysis was restricted to 3343 participants with complete information.

### 2.4. Exposure and Outcome Measures 

The outcome variable was self-reported exposure to second-hand smoke in public places. For indoor exposure, a participant was defined as exposed if he/she responded yes to the question: “Did anyone smoke in your presence, inside any enclosed public place, other than your home (such as school, shops, restaurants, police stations, hospitals, public institutions/offices, shopping malls, video clubs/movie theatres etc.) during the past 30 days?” A similar question relating to outdoor public places was asked: “Did anyone smoke in your presence, at any outdoor public place other than your home (such as car park, football field, markets, bantabas, wrestling arenas) during the past 30 days?”

The exposure variables were demographic variables including age, gender, ethnicity, residence, education, marital status, and household income. We also included awareness of regulation on smoking in public places which was assessed by asking the following question: “Are you aware of the law prohibiting smoking in public places in The Gambia?”

### 2.5. Data Management and Analysis

Immediately after the data collection, the data were cleaned, coded, and analysed using Stata 16. Descriptive statistics were used to describe the socio-demographic characteristics of the respondents, the level of compliance with smoke-free regulations and the prevalence of exposure in both indoor and outdoor public places. We estimated the prevalence of exposure to second-hand smoke in both indoor and outdoor public places by our variables of interests as highlighted in Section 2.4. Multivariable logistic regression analysis was performed to determine the sociodemographic factors associated with exposure to second-hand smoke in indoor and outdoor public places. We did not conduct multiple imputation for the missing data and hence conducted complete case analysis in all our regression models. Due to inherent difference in policy implications, three separate models for indoor, outdoor, and indoor and/or outdoor public places were run. We used confidence intervals and *p*-values in both the descriptive and multivariable regression analysis to determine level of significance.

## 3. Results 

Out of the 8856 sampled participants, 4547 responded to the survey while 16 declined to participate. The rest were either absent at the time of our visits or were not reached due to the early suspension of fieldwork activities because of COVID-19. Our analysis was restricted to 3343 individuals with complete information on the key variables. The mean age was 34.6 ± 14.5 years; more than two thirds (64.9%) of the respondents were females, 42.0% lived in urban areas, 48.9% in rural areas and 9.1% in semi-urban areas (Table 1). Most of the participants (64.9%) were married and only 10.7% had senior secondary or a higher level of education (Table 1). 

The prevalence of smoking was 5.2% and significantly higher among males compared with females (14.7% vs. 0.2%, *p* < 0.001). Awareness of the regulation that prohibits smoking in public places was moderate, 62.7%, and significantly higher among current smokers than in non-smokers (85.3% vs. 61.4%, *p* < 0.001). Half of the participants (50.9%) were not aware of the regulation that protects children from exposure to tobacco, including sale of tobacco to minors. Almost all respondents (98.1%) supported the law that prohibits smoking in public places. 

### 3.1. The Prevalence of Exposure to Second-Hand Smoke

**Figure 1:**
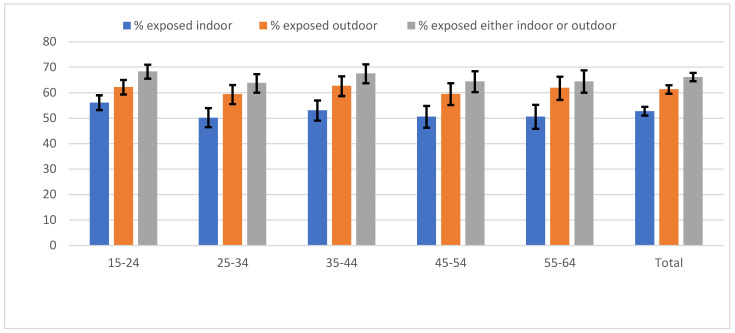
Exposure to second-hand smoke by age. N.B.: The vertical lines on top of the bars represent the 95% Confidence Intervals.

**Figure 2:**
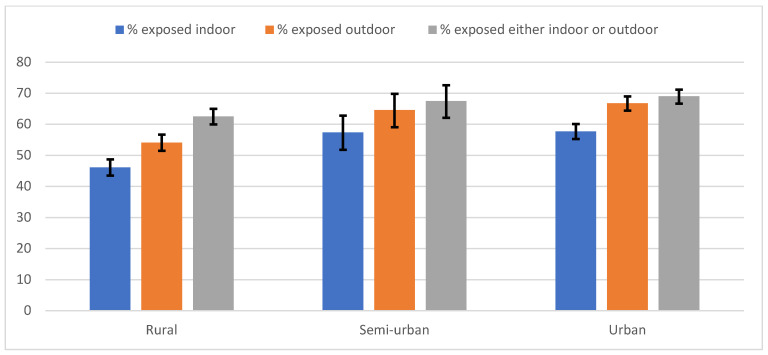
Exposure to second-hand smoke by residence. N.B.: The vertical lines on top of the bars represent the 95% Confidence Intervals.

The prevalence of exposure to second-hand cigarette smoking was higher in outdoor (61.3%) than indoor (52.8%) public places. Overall, 66.1% of the participants were exposed, with little variation by age (Figure 1). Levels of exposure were significantly higher in males than in females in all settings (indoor exposure 63.7% in males vs. 46.9% in females, *p* < 0.001; outdoor exposure: 75.6% in males vs. 53.5% in females, *p* < 0.001). In addition, exposure was higher among urban than rural residents, both indoors (46.1% vs. 57.7%, *p* < 0.001) and outdoors (54.1% vs. 66.8, *p* < 0.001) (Figure 2). 

### 3.2. Factors Associated with Exposure to Second-Hand Smoke in Indoor and Outdoor Public Places

Gender, ethnicity, residence, education, income level and level of awareness of the regulation on smoking in public were strongly associated with exposure to second-hand smoke in public places (Table 2). In model I (exposure to second-hand smoking in indoor public places), males were significantly more likely to be exposed than females (AOR = 1.78 95% CI: 1.51–2.10). Moreover, urban residents were almost three times more likely to be exposed than rural residents (AOR = 2.68, CI: 2.24–3.21). Those with higher education and those from households with higher income were less likely to be exposed in indoor public places (Table 2). The odds of exposure to second-hand smoke were significantly higher among those unaware of smoking regulations (AOR = 1.46. CI: 1.25–1.71). Models II (outdoor public places) and III (both indoor and outdoor) produced similar results. 

## 4. Discussion

Exposure to second-hand smoke in public places, despite the existing tobacco control regulations that prohibit smoking in public, remains high in The Gambia. Awareness of such regulations was moderate while it was low for the regulations protecting children. This can serve as a barrier to the implementation of tobacco control regulations. Interestingly, most respondents, including smokers, supported the law against smoking in public places and other smoke-free regulations. The factors strongly associated with exposure to second-hand smoke in both indoor and outdoor public places included male gender, lower education, lower household income, urban residence, ethnicity, and lack of awareness of smoke-free regulations.

Exposure to tobacco smoke was high and, although previous surveys focused on different age groups [3,4], exposure was similarly high in this study. A cross-sectional survey in secondary schools in The Gambia found that 59.2% of those 12–20 years old were exposed to second-hand smoke in indoor public places and 61.4% in outdoor public places [4]. Preliminary analysis of the 2017 GYTS revealed that 61.8% of students (13–15 years old) were exposed to second-hand smoke in enclosed public places [3]. In our study, women were much less exposed in both indoor and outdoor public places, a finding that is similar to results from South Africa where females were less likely to be exposed in all locations except their homes [12]. In India, males were also more likely to be exposed in public places, but females were more likely to be exposed at home [13]. 

In a previous study in The Gambia, higher maternal education and living with parents was significantly associated with lower risks of exposure while higher level of education among fathers was associated with increased odds of exposure in public places [4], suggesting highly educated mothers protected their children from exposure to cigarette smoke. In Farafenni, higher education was also a strong protector of being exposed to second-hand smoke, suggesting that increasing public awareness is a good strategy. Our findings also concur with a study in Mongolia and China where among non-smoking women, those with a higher education were less likely to be exposed to second-hand tobacco smoke compared to those with a lower level of education [12].

Individuals with a lower income were more likely to be exposed to tobacco smoke in both indoor and outdoor public places. Lower socio-economic status was associated with exposure to second-hand smoke in households in the Republic of Korea [14]. A study on exposure to domestic second-hand smoke in 15 low-and-middle-income countries reported that exposure to second-hand smoke, in most countries, was higher among the socio-economically disadvantaged [15]. These findings on socio-economic status, fairly consistent in several studies, including the current research, reflect the greater risk of second-hand smoke exposure among the less affluent because they are more likely to work or live in settings with smokers and, for public places, poorer enforcement of existing regulations. 

Women were less likely to be exposed to second-hand smoke than men. As suggested in the literature, women have a very important role in protecting themselves, children, and other family members from exposure to cigarette smoke [16]. They may also be less likely to visit public places (such as workplaces) where smokers are present. Women can be powerful advocates for health protection measures and should be involved in tobacco control at local and national level. The Ministry of Health of The Gambia has a long-standing tradition of using organized women groups and traditional communicators (most of them women) in transmitting key health messages to the population. This could be further strengthened and integrated as part of the national campaign to sensitize communities on the dangers of second-hand tobacco smoke and the tobacco control regulations. 

Education was a strong protector of being exposed to second-hand smoke which may suggest that measures raising awareness may have an important role to play, although it is also likely that more highly educated individuals are less likely to be living or working with other smokers. The results of the multivariable regression analysis show that non-smokers unaware of the regulations are more likely to be exposed to cigarette smoke in public places. Our findings also suggest that those with a lower level of education are more likely to be exposed to cigarette smoke in public places compared with participants with a high school or college/university education. As people’s knowledge of and belief in the adverse health effects of tobacco use increase, the likelihood of using tobacco decreases and their support for protective policies increases [17]. Members of a well-informed society are also more likely to take up and support policies for a tobacco-free environment. Therefore, the Ministry of Health and partners should strengthen their community sensitization programs to raise awareness of smoke-free regulations. 

### Strengths and Limitations

This is the first study on exposure to second-hand smoke in public places among both adolescents and adults in The Gambia. The study has identified both the population subgroups at a higher risk of exposure and settings where these subgroups are being exposed, and thus can be used to guide policy and enforcement efforts. These research findings can be used to inform the development of effective policies and strategies for the control of tobacco and non-communicable diseases in The Gambia, which can be adapted by other low- and middle-income countries. They can also be used as a baseline to monitor, over time, compliance with the smoke-free regulations. 

There are some limitations, including the low male response rate, mainly due to high seasonal migration, which may have underestimated overall exposure as men are more likely to smoke and to be exposed to environmental tobacco smoke. The early suspension of field activities due to the COVID-19 pandemic is another limitation as the final sample size was smaller than expected. In addition, the study was based on survey interview data and all the information on exposure to second-hand tobacco smoke was self-reported. An objective measurement of exposure to second-hand smoke, e.g., urine and/or blood analysis to assess cotinine (nicotine metabolite) was not performed. Participants can therefore under report or over report their exposure status. Future studies in The Gambia and in other African countries should explore objective measurements of exposure. 

## 5. Conclusions

Our findings illustrate that exposure of both smokers and non-smokers to second-hand smoke is high in The Gambia despite smoke-free regulations, indicating there are still gaps in tobacco control, especially on the implementation of smoke-free laws. People aware of the smoke-free regulations and women with a higher level of education were less likely to be exposed to second-hand smoke in both indoor and outdoor public places. The Ministry of Health and its partners, including the members of the Tobacco Control Committee, should continue to strengthen their advocacy and sensitization programs to ensure smoke-free regulations are fully implemented and enforced.

## Figures and Tables

**Table 1:** Characteristics of study participants (*n* = 3343).

Variable	N	%
Age group		
15–24	1101	32.9
25–34	679	20.3
35–44	616	18.4
45–54	514	15.4
55–64	433	13.0
Mean age	34.6 ± 14.5	
Sex		
Male	1175	35.2
Female	2168	64.9
Residence		
Rural	1634	48.9
Semi-urban	305	9.1
Urban	1404	42.0
Marital status		
Single (Never married)	989	29.6
Married	2222	66.5
Divorced/Separated	38	1.1
Widowed	94	2.8
Ethnicity		
Wollof	1569	46.9
Fula	716	21.4
Mandinka	920	27.5
Others	138	4.1
Education		
No formal education	284	8.5
Lower Basic	288	8.6
Upper Basic/Jun Sec	384	11.5
Senior Sec/College/Uni	358	10.7
Madrassa	655	19.6
Quranic school	1349	40.4
Senegalese	25	0.8
Household income (in Dalasi)		
Under 10,000	640	19.1
10,000–19,999	508	15.2
20,000–29,999	383	11.5
30,000–39,999	266	8.0
40,000–50,000	722	21.6
Do not know	824	24.7
Current smoking		
Yes	177	5.2
No	3166	94.7
Awareness of smoking in public regulations		
Aware	2095	62.7
Not aware	1248	37.3

**Table 2:** Factors associated with exposure to second-hand smoke in public places (*n* = 3343).

	Model I Indoor Public Places		Model II Outdoor Public Places		Model III Indoor and/or Outdoor Public Places	
	OR	AOR	OR	AOR	OR	AOR
Age group						
15–24	Reference	Reference	Reference	Reference	Reference	Reference
25–34	1.20(1.03–1.41) *	1.05(0.81–1.35)	1.12(0.95–1.31)	1.04(0.81–1.35)	1.13(0.95–1.34)	1.05 (0.81–1.37)
35–44	1.02(0.86–1.21)	0.78(0.59–1.04)	0.92(0.77–1.10)	0.78(0.59–1.04)	0.92(0.77–1.11)	0.74(0.55–1.00)
45–54	1.18(0.98–1.41)	0.87(0.64–1.18)	1.06(0.87–1.28)	0.90(0.67–1.23)	1.13(0.93–1.37)	0.88(0.64–1.20)
55–64	1.18(0.97–1.44)	0.95 (0.69–1.31)	0.97(0.79–1.19)	0.90(0.65–1.25)	1.11(0.90–1.37)	0.95(0.68–1.33)
Sex						
Female	Reference	Reference	Reference	Reference	Reference	Reference
Male	2.02(1.78–2.29) ***	1.78(1.51–2.10) ***	2.62(2.29–3.01) ***	2.40(2.02–2.86) ***	2.78(2.40–3.21) ***	2.49 (2.08–2.99) ***
Residence						
Rural	Reference	Reference			Reference	Reference
Semi–urban	1.13(0.92–1.38)	0.95(0.72–1.24)	1.10(0.89–1.35)	1.09(0.82–1.45)	1.02(0.82–1.26)	1.04 (0.78–2.25)
Urban	1.44(1.28–1.63)	2.68 (2.24–3.21) ***	1.55(1.37–1.76) ***	2.34(1.96–2.80) ***	1.23(1.08–1.40) **	1.88 (1.56–2.25) ***
Marital status						
Single	Reference	Reference	Reference	Reference	Reference	Reference
Married	1.41(1.23–1.61) ***	1.13(0.89–1.45)	1.27(1.11–1.46) ***	1.05(0.81–1.35)	1.44(1.25–1.66) ***	1.09 (0.84–1.41)
Divorced/Separated	1.76(1.05–2.96) *	1.64(0.79–3.39)	1.50(0.89–2.52)	1.00(0.50–2.02)	1.92(0.91.13–3.23) *	1.48(0.73–2.99)
Widowed	2.26(1.55–3.31) ***	1.13(0.67–1.91)	1.98(1.37–2.87) ***	1.28(0.77–2.15)	2.38(1.64–3.46) ***	1.35(0.80–2.27)
Ethnicity						
Wollof	Reference	Reference	Reference	Reference	Reference	Reference
Fula	1.15(0.98–1.34)	1.28(1.06–1.55) **	1.02(0.88–1.20)	1.16(0.96–1.41)	1.02(0.87–1.19)	1.16(0.95–1.41)
Mandinka	0.44(0.38–0.51) ***	0.43(0.36–0.52) ***	0.72(0.63–0.84) **	0.73(0.60–0.89) **	0.56(0.48–0.66) ***	0.62(0.50–0.75) ***
Others	0.49(0.36–0.66) ***	0.53(0.36–0.78) ***	0.56(0.40–0.77) **	0.60(0.40–0.90) **	0.48(0.34–0.68) ***	0.57(0.37–0.88) *
Education						
No formal education	Reference	Reference	Reference	Reference	Reference	Reference
Lower Basic	0.55(0.41–0.75) ***	0.62 (0.43–0.91) *	0.64(0.47–0.88) **	0.67(0.46–0.98) *	0.61(0.44–0.84) **	0.67(0.45–0.99) *
Upper basic/Jun Sec	0.57(0.43–0.75) ***	0.58(0.41–0.83) **	0.70(0.52–0.93) *	0.68(0.48–0.96) *	0.61(0.44–82) ***	0.60(0.42–0.88) **
Senior Sec/College/Uni	0.65(0.49–0.86) ***	0.63(0.44–0.89) **	0.60(0.45–0.81) **	0.57(0.40–0.81) **	0.57(0.42–0.78) ***	0.58(0.40–0.84) **
Madrassa	0.96(0.74–1.24)	1.08(0.79–1.47)	0.91(0.75–1.21)	0.92(0.67–1.28)	1.03(0.79–1.35)	1.01(0.74–1.40)
Quranic school	1.16(0.92–1.47)	1.27(0.96–1.69)	0.95(0.75–1.21)	0.99(0.75–1.31)	1.09(0.85–1.39)	1.10(0.83–1.46)
Senegalese	0.88(0.43–1.82)	0.70(0.30–1.66)	0.62(0.29–1.36)	0.59(0.251.42)	0.70(0.32–1.57)	0.71(0.29–1.72)
Household income						
Under 10,000	Reference	Reference	Reference	Reference	Reference	Reference
10,000–19,999	1.17(0.94–1.45)	1.54 (1.20–1.99) ***	1.04(0.84–1.30)	1.36(1.05–1.75) *	1.06(0.85–1.32)	1.24(0.96–1.60)
20,000–29,999	0.88(0.70–1.13)	1.16(0.89–1.53)	0.84(0.66–1.07)	1.02(0.78–1.35)	0.83(0.65–1.07)	0.96(0.73–1.28)
30,000–39,999	0.90(0.69–1.18)	1.09(0.80–1.49)	0.69(0.52–0.91) **	0.72(0.53–1.00)	0.67(0.50–0.90) **	0.68(0.49–0.95) *
40,000–50,000	0.86(0.70–1.04)	0.82(0.65–1.04)	0.80(0.66–0.98) *	0.71(0.56–0.90) **	0.73(0.60–0.90) ***	0.70(0.55–0.90) ***
Do not know	0.84(0.70–1.02)	0.93(0.75–1.17)	0.80(0.66–0.98)	0.82(0.65–1.02)	0.88(0.37–2.15)	0.73(0.58–0.92) **
Awareness of smoking in public regulation						
Aware	Reference	Reference	Reference	Reference	Reference	Reference
Not aware	1.68(1.49–1.90) ***	1.46(1.25–1.71) ***	1.76(1.56–1.99) ***	1.47(1.26–1.73) ***	1.80(1.59–2.04) ***	1.48(1.26–1.73) ***

* *p* < 0.05, ** *p* ≤ 0.01, *** *p* ≤ 0.001. OR = Unadjusted odds ratio. AOR = Adjusted odds ratio. Reference = Reference category in regression n analysis.

## Data Availability

Data reported in this study are from an ongoing longitudinal study, but the data is not yet publicly available.
